# Steroidogenic differentiation of human amniotic membrane-derived mesenchymal stem cells into a progesterone-/androgen-producing cell lineage by SF-1 and an estrogen-producing cell lineage by WT1−KTS

**DOI:** 10.3389/fendo.2024.1410433

**Published:** 2024-09-18

**Authors:** Yumiko Miyazaki, Makoto Orisaka, Yuko Fujita, Tetsuya Mizutani, Takashi Yazawa, Yoshio Yoshida

**Affiliations:** ^1^ Department of Obstetrics and Gynecology, Faculty of Medical Sciences, University of Fukui, Fukui, Japan; ^2^ Department of Nursing, Faculty of Nursing and Welfare Sciences, Fukui Prefectural University, Fukui, Japan; ^3^ Department of Biochemistry, Asahikawa Medical University, Hokkaido, Japan

**Keywords:** cell differentiation, gonadal steroid hormones, mesenchymal stem cells, steroidogenic factor 1, Wilms’ tumor 1

## Abstract

**Background:**

Sex steroid hormones, primarily synthesized by gonadal somatic cells, are pivotal for sexual development and reproduction. Mice studies have shown that two transcription factors, steroidogenic factor 1 (SF-1) and Wilms’ tumor 1 (WT1), are involved in gonadal development. However, their role in human gonadal somatic differentiation remains unclear. We therefore aimed to investigate the roles of SF-1 and WT1 in human gonadal steroidogenic cell differentiation.

**Methods:**

Using a transient lentivirus-mediated gene expression system, we assessed the effects of SF-1 and WT1 expression on the steroidogenic potential of human amniotic membrane-derived mesenchymal stem cells (hAmMSCs).

**Results:**

SF-1 and WT1−KTS, a splice variant of WT1, played distinct roles in human steroidogenic differentiation of hAmMSCs. SF-1 induced hAmMSC differentiation into progesterone- and androgen-producing cell lineages, whereas WT1−KTS promoted hAmMSC differentiation into estrogen-producing cell lineages.

**Conclusion:**

Our findings revealed that SF-1 and WT1−KTS play important roles in human gonadal steroidogenic cell differentiation, especially during ovarian development. These findings may pave the way for future studies on human ovarian differentiation and development.

## 1 Introduction

Sex steroid hormones, including progesterone, androgens, and estrogen, play critical roles in sexual development, reproduction, and body homeostasis. They are mainly synthesized by gonadal somatic cells, such as the Sertoli and Leydig cells in the male testis, granulosa and theca cells in the female ovary, and cortical cells in the adrenal gland. The origin of these steroidogenic cells is thought to be the adrenogonadal primordium (AGP) in the genital ridge (1, 2). Mice studies have shown that two transcription factors, steroidogenic factor 1 (SF-1) and Wilms’ tumor 1 (WT1), are essential for AGP development (3, 4). SF-1 and WT1 are expressed in the undifferentiated genital ridge of human fetuses at 6 weeks of pregnancy (5), suggesting that these transcription factors may be involved in steroidogenic cell differentiation in humans as well.

*SF-1* encodes an orphan nuclear receptor, SF-1. SF-1 is expressed not only in the fetal genital ridge but also in adult testicular Leydig cells, ovarian granulosa and theca cells, and adrenal cortical cells (6, 7). SF-1-null mice lacked adrenal glands and gonads, indicating that SF-1 is indispensable for adrenal and gonadal development (8). Furthermore, SF-1 has been shown to induce the expression of various steroidogenic enzymes by binding to shared promotor elements and is believed to be a master regulator of steroid hormone production (6, 9).

*WT1* encodes a zinc finger nuclear transcription factor, WT1 (3). WT1 is expressed not only in the fetal genital ridge but also in adult testicular Sertoli cells and ovarian granulosa cells (7). WT1 has two major alternatively spliced isoforms defined by the presence or absence of three amino acids (lysine, threonine, and serine [KTS]) between the third and fourth zinc fingers. These isoforms are referred to as WT1+KTS and WT1−KTS, respectively. WT1+KTS and WT1−KTS have different biochemical and biological properties (10). WTS+KTS binds to RNA and is involved in RNA metabolism (11); in contrast, WT1−KTS binds to DNA and directly regulates gene expression (12, 13). A recent study suggests that WT1−KTS may be an ovarian-determining factor in female mice, similar to the sex-determining region Y (*SRY*) gene, which is the testis-determining gene in males (14).

Mesenchymal stem cells (MSCs) are non-hematopoietic, undifferentiated, fibroblast-like cells that have multipotent differentiation capacity (15). We previously induced the differentiation of human bone marrow-derived MSCs into steroidogenic cells by forced expression of SF-1 (16, 17). However, harvesting MSCs from the bone marrow requires invasive bone marrow aspiration. In contrast, fetal appendages, such as the placenta and umbilical cord, are usually discarded after birth and are one of the richest sources of MSCs.

Nevertheless, the mechanisms underlying the differentiation of gonadal somatic cells in humans are not fully understood. Thus, in the present study, we aimed to investigate the role of SF-1 and WT1 in human steroidogenic cell differentiation using human amniotic membrane-derived MSCs (hAmMSCs).

## 2 Materials and methods

### 2.1 Isolation of MSCs from human amniotic membrane

**Figure 1:**
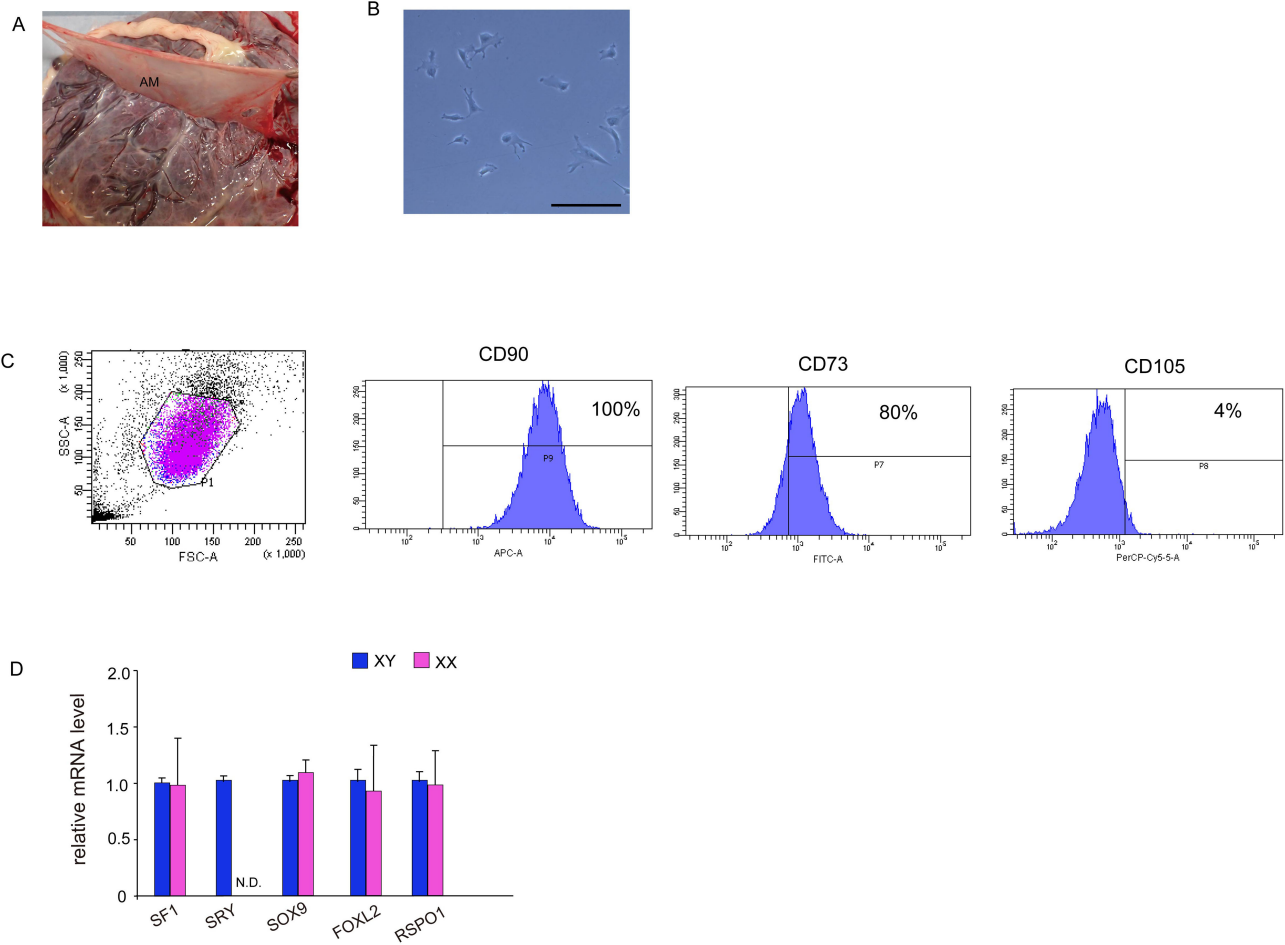
Isolation and characterization of MSCs from human amniotic membrane. **(A)** Harvesting of human amniotic membrane, a thin translucent layer attached to the chorionic membrane. **(B)** Phase contrast images taken 24 h after isolation. Culture cells display a fibroblast-like morphology and spindle shape similar to that displayed by human MSCs. Scale bar, 50 μm. **(C)** Fluorescence-activated cell sorting analysis. hAmMSCs were separated using antibodies for positive marker genes (CD90, CD73, and CD105) and negative marker genes (CD45, CD34, CD11b, CD79A, and HLA-DR). Almost all cells were CD90-positive, 80% were CD73-positive, and only 4% were CD105-positive. **(D)** The expression of several representative genes involved in gonadal development in hAmMSCs. *SRY* expression was only observed in hAmMSCs derived from male babies. Data are presented as the mean ± SD from three independent experiments. N.D., not detectable. MSCs, mesenchymal stem cells; hAmMSCs, human amniotic membrane-derived mesenchymal stem cells; SSC, side-scattered; FSC, forward scattered; SRY, sex-determining region Y.

This study was approved by the Institutional Ethics Committee of the University of Fukui (approval number 20170139), and consent was obtained from each donor. Full-term placentas (n = 19) were collected from healthy donor mothers who underwent caesarean section deliveries at the University of Fukui Hospital from March 2018 to August 2021. Among them, 7 samples were from pregnancies with male offspring, and 12 samples were from pregnancies with female offspring. The amniotic membrane from each specimen was mechanically separated from the underlying chorion via blunt dissection (**Figure 1A**). Cells were isolated from the amniotic membrane near the umbilical cord. The membrane sections were minced and rinsed with phosphate-buffered saline (PBS) to remove blood. The fragments were enzymatically digested in two steps: (1) incubation with 0.125% trypsin and 0.5 mM ethylenediaminetetraacetic acid (EDTA) solution at 37 °C for 30 min, and (2) treatment with 100 U/mL collagenase type II (Thermo Fisher Scientific, Waltham, MA, USA) and 3 mM calcium chloride in Dulbecco’s modified Eagle’s medium (Wako, Pure Chemical Industries, Osaka, Japan) at 37°C for 120 min in a shaker, followed by washing with PBS. The cell suspension was seeded into 10-cm dishes in Mesenchymal Stem Cell Growth Medium (Promo Cell, Heidelberg, Germany) containing 100 U/mL penicillin and 100 mg/mL streptomycin. Cells were cultured at 37°C in a 5% CO_2_ atmosphere, and the medium was replaced every 3 days.

After 5–7 days of culture, non-adherent cells were removed, and adherent cells were detached with 0.05% trypsin and EDTA and washed once with PBS containing 1% bovine serum albumin. The cell suspensions were sorted using the Human Mesenchymal Stem Cell Validation Flow Kit (R&D Systems, Abingdon, UK) and stained with the following antibodies: APC-conjugated CD90 antibody, FITC-conjugated CD73 antibody, PerCP-conjugated CD105 antibody, and phycoerythrin-conjugated negative markers (CD45, CD34, CD11b, CD79A, and HLA-DR). The cells were incubated in the dark at room temperature for 30 min. Cell sorting was carried out using a FACSAria II (BD Biosciences, San Jose, CA, USA). The sorted and isolated cells were used for subsequent *in vitro* differentiation and transfections.

### 2.2 *In vitro* differentiation test

To confirm the presence of MSCs in the culture of amniotic membrane-derived cells (hAmMSCs), the differentiation capacity of the cells into bone, cartilage, and fat cells was analyzed. To induce adipogenic differentiation, the amniotic membrane-derived cells were seeded in Mesenchymal Stem Cell Adipogenic Differentiation Medium (PromoCell) at a density of 2×10^4^ cells/cm^2^ and cultured for 4 weeks. Medium replacements were carried out twice weekly. After treatment, differentiation to adipose tissues was confirmed by staining with Oil Red O. To induce osteogenic differentiation, the amniotic membrane-derived cells were seeded in Mesenchymal Stem Cell Osteogenic Differentiation Medium (PromoCell) at a density of 2×10^4^ cells/cm^2^ and cultured for 1 week, replacing the medium every 3 days. After treatment, differentiation to bone tissue was confirmed by staining the bone matrix with 2% Alizarin Red S solution. To induce chondrogenic differentiation, 2.5×10^5^ amniotic membrane-derived cells were transferred into 15-mL polypropylene tubes and centrifuged at 100 ×*g* for 5 min to form pellets, followed by culturing in Mesenchymal Stem Cell Chondrogenic Differentiation Medium (PromoCell) for 4 weeks. Medium replacements were carried out twice weekly. After treatment, differentiation to cartilage tissue was confirmed by staining sulfated proteoglycans with Alcian blue.

### 2.3 Lentivirus preparation and infection

Green fluorescent protein (GFP), SF-1, WT1+KTS, and WT1−KTS were transiently expressed in the hAmMSCs via lentivirus-mediated gene transfection. As a control, these genes were also expressed in normal human dermal fibroblasts (C-12302, Promo cell). The lentivirus packaging plasmids were kindly provided by Dr. Yanase, Fukuoka University (Fukuoka, Japan) (18). The packaging cell line, Lenti-X 293T Cell Line (Takara, Tokyo, Japan), was transfected with the lentiviral His plasmids using the Trans IT-293T transfection reagent (Takara) in Opti-MEM™. The supernatant was concentrated by centrifugation, and the virus solution was stored at −80 °C until use. The day before infection, hAmMSCs or normal human dermal fibroblasts were seeded into 96-well plates at a density of 2–3×10^4^ cells/cm^2^. After 12 h, the culture medium was replaced, and 10 µL of infection solution containing 8 µg/mL polybrene (Sigma-Aldrich, St. Louis, MO) was added; 48 h after infection, whole-cell lysates were collected for assay.

### 2.4 Steroid hormone assay

After treatments, the conditioned media were collected and briefly centrifuged to sediment any dead cells. The supernatants were immediately frozen at −80°C until further use. According to the manufacturer’s instructions, steroid hormone concentrations for each sample were measured using EIA kits for aldosterone, cortisol, progesterone, testosterone, estradiol (Cayman Chemical, Ann Arbor, MI), androstenedione (OriGene Rockville, MD), dehydroepiandrosterone (DHEA) (IBL international, Hamburg, Germany), total cholesterol (Finetest, Hubei, China), and pregnenolone (Abnova, Taipei, Taiwan). To analyze estradiol biosynthesis in WT1−KTS-induced hAmMSCs, 10^−7^ to 10^−6^ M testosterone (Sigma Aldrich) was added to the media as an aromatizable substrate. Each experiment was performed at least twice over three independent experiments. The intra-assay and inter-assay coefficients of variation (CV) are shown in **Supplementary Table 1**.

### 2.5 Real-time reverse transcription polymerase chain reaction

The transfected hAmMSCs were washed with PBS, and total RNA was extracted using SuperPrepII Cell Lysis & RT Kit for qPCR (Toyobo), followed by reverse transcription polymerase chain reaction (RT-PCR) using SYBR Green PCR Master Mix (Thermo Fisher Scientific, Waltham, MA) on the StepOnePlus™ RT-PCR system (Thermo Fisher Scientific). The gene-specific primers for real-time RT-PCR are shown in **Table 1**. Owing to the high sequence homology, detecting the WT1 isoforms (i.e. WT1+KTS and WT1−KTS) by real-time PCR was problematic. Therefore, touch-down PCR was performed to increase the sensitivity and specificity for WT1+KTS and WT1−KTS (19). The results were analyzed using the 2^−ΔΔCT^ method. Three independent experiments were replicated, and glyceraldehyde 3-phosphate dehydrogenase was used as an internal control gene.

**Table 1:** Human primer pairs used to assess the steady-state mRNA level of target genes.

Gene	GenBank Accession No.		Primer pairs	Product size (bp)
*SF-1*	NM_004959	Forward Reverse	GACGAGGACCTGGACGAGCTGTG GATCTTGCAGCTCTGGCTCTCGGTG	156
*StAR*	NM_000349	Forward Reverse	CCACCCCTAGCACGTGGAT TCCTGGTCACTGTAGAGAGTCTCTTC	89
*SRY*	NM_003140	Forward Reverse	AAGATGCTGCCGAAGAATTG TCTTGAGTGTGTGGCTTTCG	120
*SOX9*	NM_000346	Forward Reverse	AGCGAACGCACATCAAGAC CTGTAGGCGATCTGTTGGGG	85
*CYP11A1*	NM_000781	Forward Reverse	CAGGAGGGGTGGACACGAC AGGTTGCGTGCCATCTCATAC	64
*CYP17A1*	NM_000102	Forward Reverse	TGAGTTTGCTGTGGACAAGG TCCGAAGGGCAAATAGCTTA	163
*CYP19A1*	NM_000103	Forward Reverse	CCCTTCTGCGTCGTGTCAT GATTTTAACCACGATAGCACTTTCG	86
*CYP21A2*	NM_047443023	Forward Reverse	TCAGGTTCTTCCCCAATCCA TCCACGATGTGATCCCTCTTC	70
*HSD3B2*	NM_000862	Forward Reverse	GCCTTCCAGACCAGAATTGAGAGA TCCTTCAAGTACAGTCAGCTTGGT	73
*HSD17B1*	NM_001330219	Forward Reverse	TTCCTGCCAGACATGAAGAGGC AGAACCGCCAGACTCTCGCATA	143
*HSD17B3*	NM_000197	Forward Reverse	TGTCATCTTTTGTAAAATCTGCTTG AGGCCATTGCCACAGAGA	78
*AMHR2*	NM_001164691	Forward Reverse	TGTGTTTCTCCCAGGTAATCCG AATGTGGTCGTGCTGTAGGC	164
*LHCGR*	NM_000233	Forward Reverse	ACACTTTATTCTTCCATGCTTGCTGAG ATTAAAAGCATCTGGTTCAGGAGCACA	111
*FSHR*	NM_000145	Forward Reverse	AACACCCATCCAAGGAATGG GGGCTAAATGACTTAGAGGGACAA	91
*AR*	NM_000044	Forward Reverse	CCTGGCTTCCGCAACTTACAC GGACTTGTGCATGCGGTACTCA	168
*ACTHR*	NM_001291911	Forward Reverse	AAGAATAAGAATCTCCAGGCACC TCCGCAGCAATCACAGACA	230
*LHX9*	NM_001410927	Forward Reverse	ACCTGCTTTGCCAAGGACGGTA TGACCATCTCCGAGGCGGAAAT	112
*FOXL2*	NM_023067	Forward Reverse	GAGTTTTTGTTGGGCCTTCA GAGGGTGAAACTTCCCCAAT	97
*WNT6*	NM_00652	Forward Reverse	GTGCAACTGCACAACAACGAGG GAAATGGAGGCAGCTTCTGCCA	127
*ADH1A2*	NM_170697	Forward Reverse	GAGTAACTCTGGAACTTGGAGGC ATGGACTCCTCCACGAAGATGC	151
*RSPO1*	NM_001242910	Forward Reverse	ACACTTCCCAGCATCTGAGACCAA TGCTGAACAGGATGGGAAGAAGGT	146
*NR0B1*	NM_000475	Forward Reverse	CCAAATGCTGGAGTCTGAACATC CCCACTGGAGTCCCTGAATGTA	120
*OSR2*	NM_001394683	Forward Reverse	TTGCTCATCCATGAGAGGACCC CCACACTCCTGACATTTGAAGGG	143
*NR2F2*	NM_001145157	Forward Reverse	TGCACGTTGACTCAGCCGAGTA AAGCACACTGAGACTTTTCCTGC	120
*LIFR*	NM_001364298	Forward Reverse	CACCTTCCAAAATAGCGAGTATGG ATGGTTCCGACCGAGACGAGTT	159
*PDGFRα*	NM_001347830	Forward Reverse	GACTTTCGCCAAAGTGGAGGAG AGCCACCGTGAGTTCAGAACGC	121
*INSL3*	NM_005543	Forward Reverse	TCTGTCCCCACTGAATCCTCCTGG GGGTTTCATGGTGCTGTGTGGC	102
*GAPDH*	NM_002046	Forward Reverse	TGCACCACCAACTGCTTAGC GGCATGGACTGTGGTCATGAG	87
*WT1(+KTS)* *WT1(−KTS)*	NM_001429031 NM_001429032	Forward Reverse Forward Reverse	GCTCAAAAGACACCAAAGGAGACA CTGAAGGGCTTTTCACTTGTTTTAC GCTCAAAAGACACCAAAGGAGACA GCT GAA GGG CTT TTC ACC TGT A	138 130

#### 2.5.1 Effect of SF-1 and WT1 on steroidogenic cell lineages

mRNA expression in hAmMSCs was analyzed to investigate the effect of SF-1 and WT1 on each steroidogenic cell lineage. In progesterone-producing cell lineages, mRNA expression of progesterone-producing factors (*StAR*, *CYP11A1*, and *HSD3B2*) was analyzed, whereas in adrenal-producing steroidogenic cell lineages, mRNA expression of adrenal-specific markers (*NR0B1*, *OSR2*, *ACTHR*, and *AR*) and adrenal steroidogenic enzymes (*CYP21A2*, *CYP11B1*, and *CYP11B2*) was analyzed. In androgen-producing cell lineages, mRNA expression of androgen-specific markers (*NR2F2*, *LIFR*, *PDGFRα*, and *LHCGR* in Leydig cells, *INSL3* in Theca cells) and androgen-producing enzymes (*CYP17A1* and *HSD17B3*) was analyzed. In estrogen-producing cell lineages, mRNA expression of estrogen-specific markers (*ALDH1A2*, *WNT6*, *FOXL2*, *AMHR2*, *FSHR* etc. in granulosa cells) (20) and estrogen-producing enzymes (*CYP19A1* and *HSD17B1*) was analyzed.

#### 2.5.2 Expression of the tissue-specific splice form of CYP19A1 in hAmMSCs

The human *CYP19A1* (aromatase) gene contains 10 tissue-specific promoters (I.1, I.2, I.2a [placenta], I.3 [adipose/breast], I.4 [skin/adipose], I.5 [fetal tissues], I.6 [bone], I.7 [endothelial/uterus], I.f [brain], and II [ovary]), that are alternatively used in various cell types. These promoters regulate aromatase expression in various tissues in a tissue-specific manner (21). The mRNA expression of these tissue-specific splice forms of *CYP19A1* in hAmMSCs was analyzed.

### 2.6 Western blot

The transfected hAmMSCs were washed with PBS and lysed using radio-immunoprecipitation assay buffer (Cell Signaling Technology, Beverly, MA). Approximately 20 µg of protein sample was electrophoresed via sodium dodecyl-sulfate polyacrylamide gel electrophoresis, transferred to a polyvinylidene difluoride membrane, and probed sequentially with primary antibodies against WT1 (1:500, NB110-60011; Novus Biologicals) and β-actin (1:1000, Cell Signaling) at 4°C overnight, followed by incubation with horseradish peroxidase-coupled secondary antibodies for 60 min at room temperature. Chemiluminescent signals were developed using Western Lightning ECL Pro (PerkinElmer, Inc.) and detected using a cooled charge-coupled device camera (LAS 4000 Mini; GE Healthcare Life Sciences).

### 2.7 Immunostaining

Immunocytochemistry was performed to investigate aromatase expression in transfected hAmMSCs. The hAmMSCs were cultured on cell culture slides (SPL Life Sciences) at a density of 2.3×10^4^ cells/well. After 48 h of lentiviral infection, the cells were washed with PBS and fixed with 4% paraformaldehyde for 20 min. The cells were then incubated with Blocking One (Nacalai Tesque, Inc, Kyoto, Japan) for 10 min at room temperature, after which they were immunolabeled with the primary antibody WT1 (1:100, NB110-60011; Novus Biologicals, Centennial, CO, USA) and anti-aromatase antibody (1:100, NBP2-61939; Novus Biologicals) for 1 h. After washing three times with PBS, the secondary antibody donkey anti-mouse Alexa 488 (1:1000, Invitrogen) was used for detection. Nuclei were stained with 4’,6-diamidino-2-phenylindole (vector). Finally, the sections were examined with an Olympus U-HGLGPS microscope (Tokyo, Japan).

### 2.8 Luciferase assay for CYP19A1 PII promoter

The CYP19A1 PII promoter (-2039/-16) was amplified via PCR and cloned into pGL4.11 luciferase vector (Promega). The vector was sequence-verified. hAmMSCs were dispensed into 24-well plates at a density of 5×10^4^ cells/well and co-transfected with 200 ng of CYP19A1 PII promoter luciferase plasmid and WT1−KTS or GFP expression plasmid (100 ng/well) using 1.2 μL of TransIT-Lenti Transfection Reagent (Mirus Bio). To correct for differences in transfection efficiency, *Renilla* luciferase reporter vector (Promega) was co-transfected as an internal control. Luciferase activity was assayed 48 h after transfection using Dual Luciferase Reporter Assay System (Promega) and measured using Luminescencer Octa AB-2270 (ATTO, Tokyo, Japan).

### 2.9 Statistical analysis

Statistical analyses were performed using GraphPad Prism 10 (GraphPad Software, San Diego, CA, USA). Differences between groups were analyzed using the t-test and one-way analysis of variance, with a p-value <0.05 considered statistically significant.

## 3 Results

### 3.1 Isolation and characterization of MSCs from human amniotic membrane

After enzymatic digestions and cell extraction from human amniotic membrane, an adherent cell population was obtained by primary culture (**Figure 1B**). To confirm the presence of MSCs, the cell surface markers were checked via flow cytometry using three positive markers (CD73, CD90, and CD105) and five negative markers (CD45, CD34, CD11b, CD79A, and HLA-DR) on cells from the second passage. CD90 and CD73 were highly expressed on the cell surface, while the positivity rate for CD105 was 4% (**Figure 1C**). The expression of negative markers was low in the cell population. These results demonstrated that 4% of adherent amniotic membrane-derived cells were hAmMSCs. Hence, for subsequent experiments, we decided to only use the homogeneous hAmMSCs that were positive for all three markers (CD73, CD90, and CD105). We investigated whether the expression of genes involved in early development of the adrenal gland (*SF-1*), testis (*SRY* and *SOX9*), and ovary (*FOXL2* and *RSPO1*) differs in hAmMSCs depending on fetal sex. No differences in gene expression by sex were observed, except that female-derived hAmMSCs did not express *SRY* (**Figure 1D**). In this study, hAmMSCs were collected and pooled from 12 female placentas for the subsequent experiments. because our main objective was to investigate the steroidogenic differentiation of ovarian somatic cells rather than that of testicular somatic cells.

**Figure 2:**
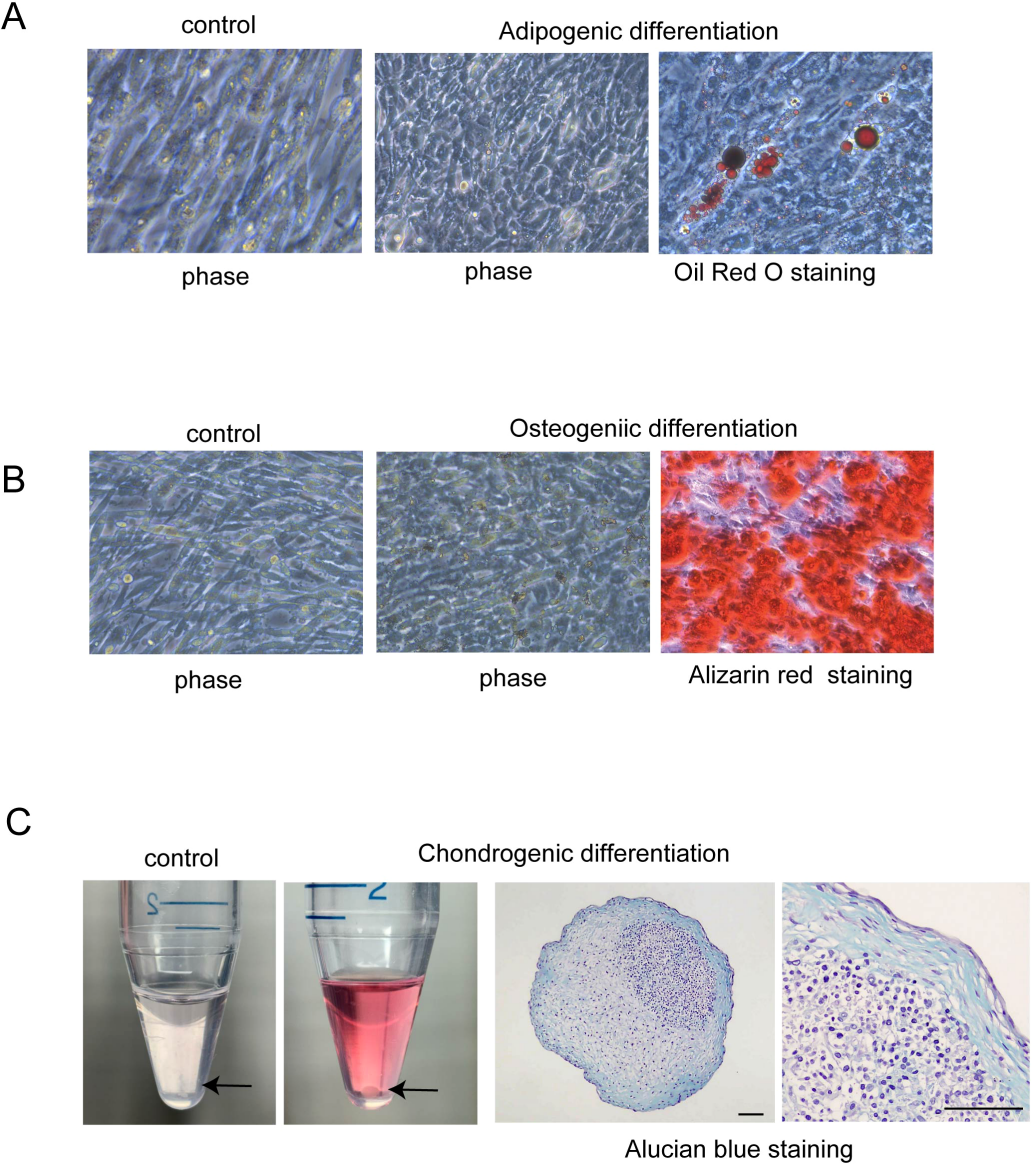
Differentiation capacity of hAmMSCs. **(A)** Adipogenic differentiation was evidenced by Oil Red O staining. Lipid vacuoles were formed in the hAmMSCs. **(B)** Osteogenic differentiation was evidenced by Alizarin Red S staining. Mineralized matrix was formed in the hAmMSCs. **(C)** The hAmMSCs cultured with chondrogenic differentiation medium formed pellets. Note that the cells did not form pellets in the control medium (RPMI 1640 plus 10% Fetal Calf Serum). Chondrogenic differentiation was evidenced by Alcian blue staining. Scale bar, 100 µm. hAmMSCs, human amniotic membrane-derived mesenchymal stem cells.

To confirm the multipotency of hAmMSCs, their differentiation capacity into fat, bone, and cartilage cells was evaluated. Oil Red O staining positively identified the lipid droplets formed in the adipogenic medium-treated hAmMSCs (**Figure 2A**). Alizarin Red S staining positively identified the mineralized matrix formed in the osteogenic medium-treated hAmMSCs (**Figure 2B**). Alcian blue staining positively identified the accumulation of sulfated proteoglycans in the chondrogenic medium-treated hAmMSCs (**Figure 2C**). These results indicate that the hAmMSCs used in this study are multipotent. We used the hAmMSCs within four passages in subsequent experiments, because MSCs age and lose their multipotency during long-term *in vitro* culture (22).

### 3.2 Gene transfection into hAmMSCs

**Figure 3:**
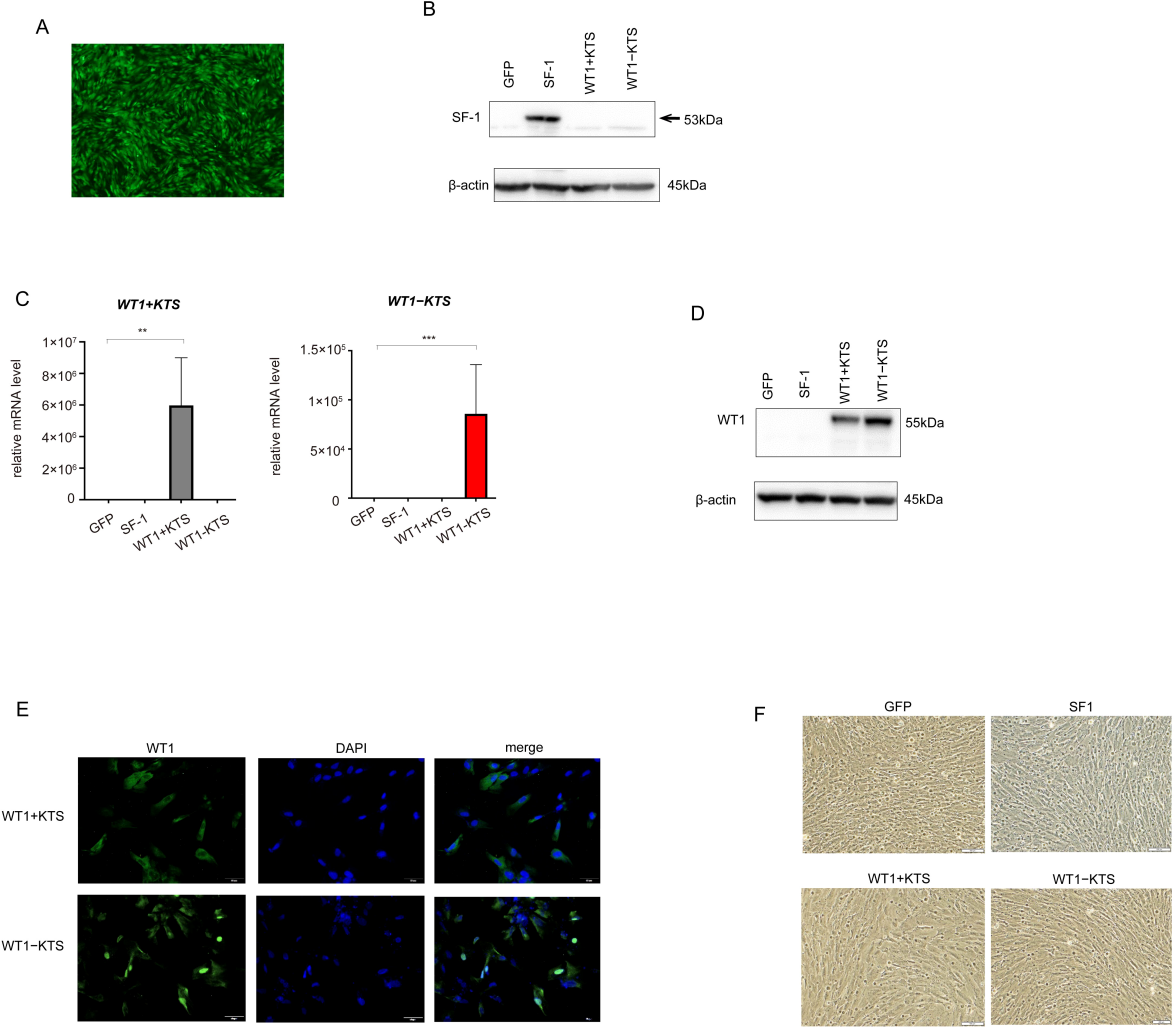
Lentivirus-mediated gene transfection into hAmMSCs. **(A)** The hAmMSCS 48 h after GFP transfection. Almost all hAmMSCs expressed GFP. **(B)** SF1 expression in hAmMSCs was confirmed via western blot analysis. **(C)** WT1+KTS and WT1−KTS mRNA expression in hAmMSCs was confirmed via quantitative PCR using isoform-specific primers. **(D)** Expression of WT1 in hAmMSCs was confirmed via western blot analysis. **(E)** WT1+KTS and WT1−KTS were mainly expressed in the cytoplasm and partially in the nucleus. **(F)** Neither the SF-1- nor WT1-transfected hAmMSCs showed any morphological changes compared with those transfected with GFP. Data are presented as the mean ± SD from three independent experiments. **, *p*<0.01, ***, *p*<0.001. hAmMSCs, human amniotic membrane-derived mesenchymal stem cells; GFP, green fluorescent protein; WT1, Wilms’ tumor 1; SF-1, steroidogenic factor 1; RT-PCR, reverse transcription polymerase chain reaction; SD, standard deviation.

GFP, SF-1, WT1+KTS, and WT1−KTS were transiently expressed in hAmMSCs via lentivirus-mediated gene transfection. After 48 h of induction, almost all hAmMSCs expressed GFP (**Figure 3A**). The expression of SF-1 in the hAmMSCs was confirmed via western blot analysis (**Figure 3B**). The mRNA expression of WT1+KTS and WT1−KTS in hAmMSCs was confirmed via quantitative PCR using isoform-specific primers (**Figure 3C**). WT1 expression in hAmMSCs was also verified through western blot analysis (**Figure 3D**). Immunohistochemical analysis indicated that WT1+KTS and WT1−KTS were mainly expressed in the cytoplasm and partly in the nucleus (**Figure 3E**). At 48 h post-induction, neither SF-1- nor WT1−KTS-transfected hAmMSCs exhibited morphological changes compared with those transfected with GFP (**Figure 3F**).

### 3.3 Gene transfection into human fibroblasts

**Figure 4:**
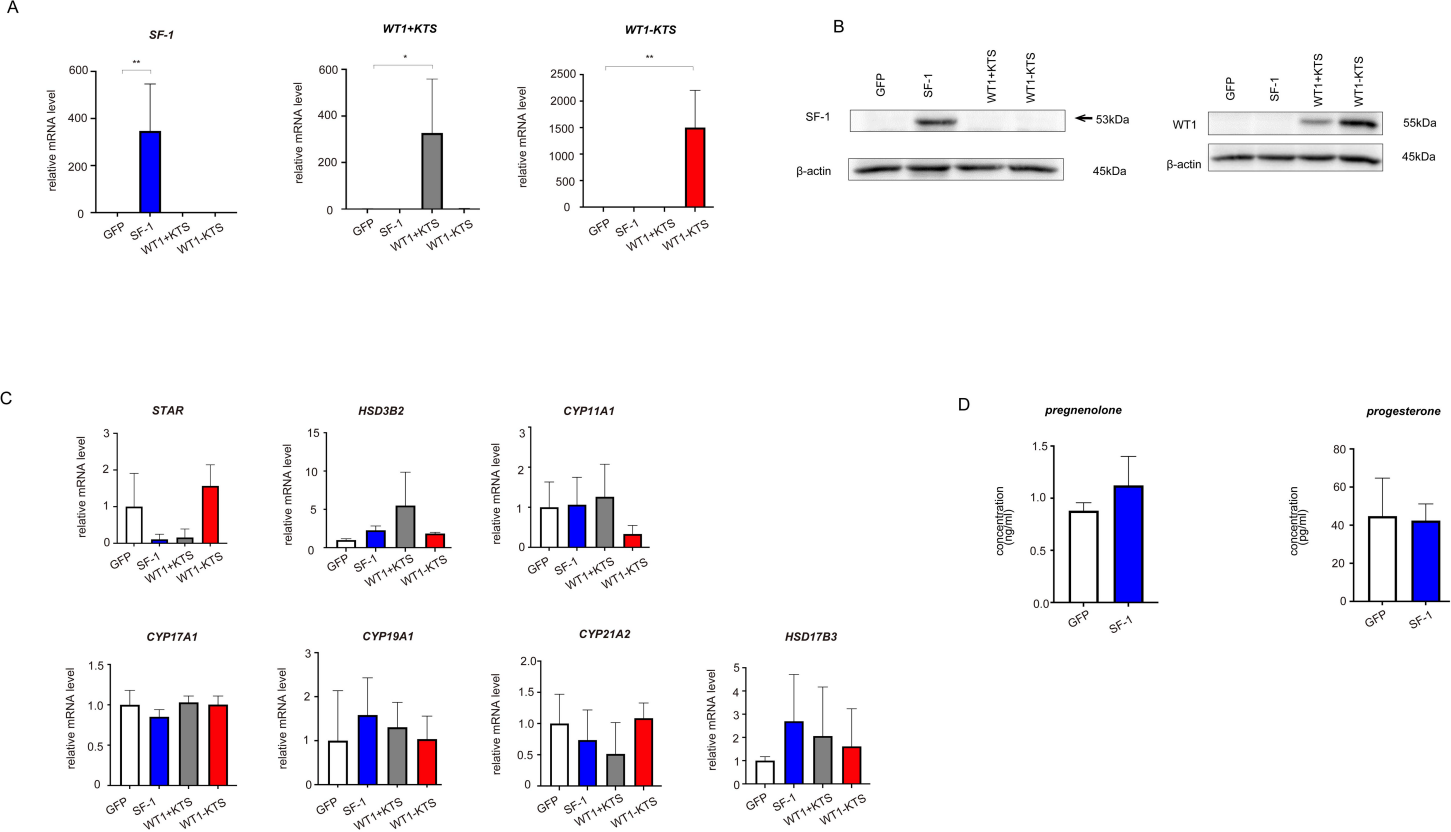
Lentivirus-mediated gene transfection into human fibroblasts. **(A)** SF-1, WT1+KTS, and WT1−KTS mRNA expression in human fibroblasts was confirmed via quantitative PCR. **(B)** SF-1 and WT1 expression in human fibroblasts was confirmed via western blot analysis. **(C)** Real-time RT-PCR analysis revealed that neither SF-1 nor WT1 transient expression affected the gene expression of steroidogenic factors in human fibroblasts. **(D)** SF-1 did not enhance pregnenolone and progesterone production by human fibroblasts. Data are presented as the mean ± SD from three independent experiments. *, p<0.05, **, p<0.01.

As a control for the multipotent hAmMSCs, differentiated cells (human fibroblasts) were transfected with GFP, SF-1, WT1+KTS, and WT1−KTS (**Figures 4A, B**) and examined whether the mRNA expression of steroidogenesis-related factors was altered. Real-time RT-PCR analysis showed that transient expression of SF-1 or WT1 did not affect the gene expression of steroidogenic factors in human fibroblasts (**Figure 4C**). SF-1 did not enhance pregnenolone and progesterone production by human fibroblasts (**Figure 4D**).

### 3.4 Effects of SF-1 and WT1 on progesterone-producing cell lineage

**Figure 5:**
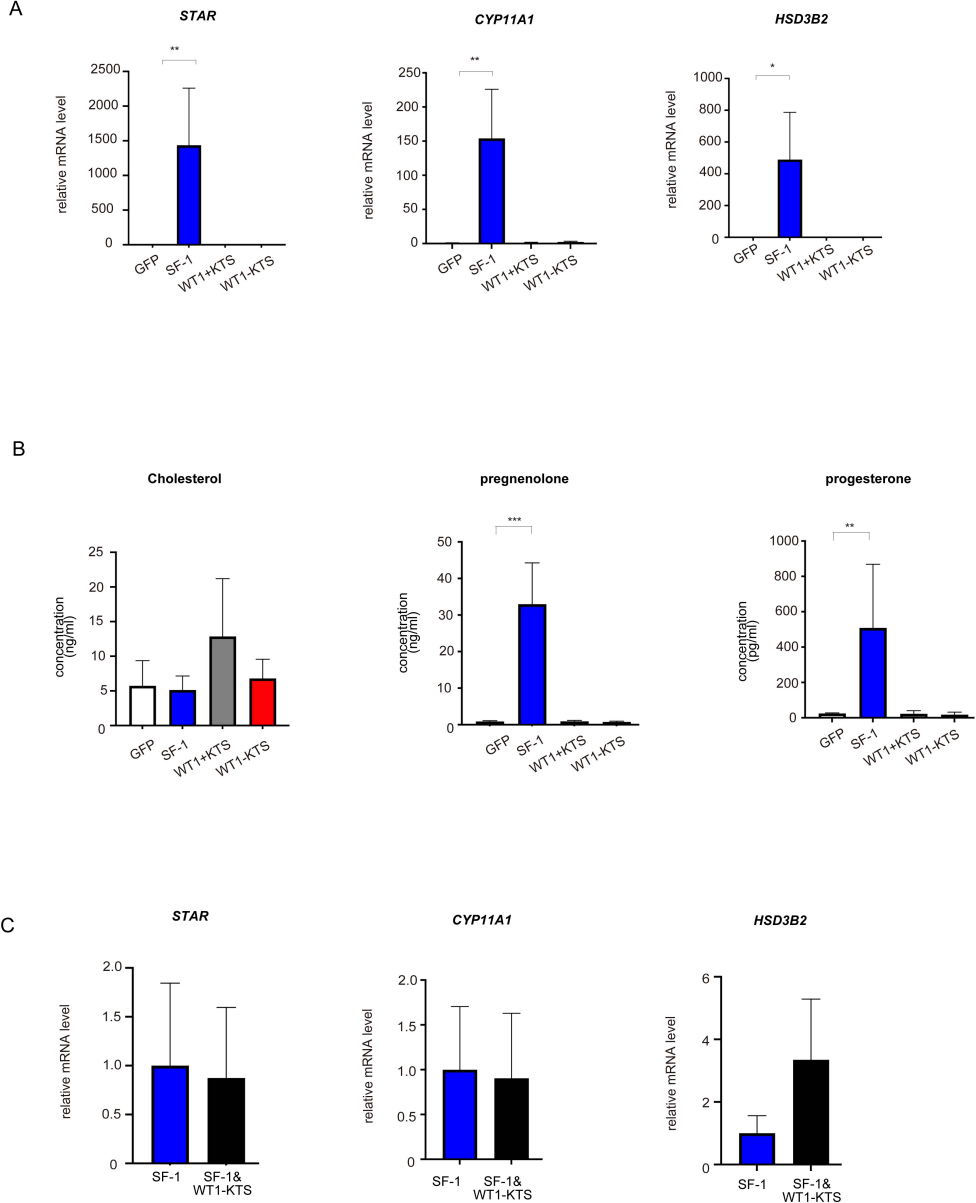
Effects of SF-1 and WT1 on progesterone-producing cell lineage. GFP, SF-1, WT1+KTS, and WT1−KTS were transiently expressed in hAmMSCs via lentivirus-mediated gene transfection and analyzed for progesterone-producing capacity. **(A)** The expression of steroidogenic genes involved in progesterone synthesis in hAmMSCs was measured via quantitative PCR. SF-1 considerably increased the expression of *StAR*, *CYP11A1*, and *HSD3B2* in hAmMSCs. In contrast, WT1+KTS and WT1−KTS did not alter the mRNA expression of progesterone-producing factors. **(B)** SF-1 significantly enhanced pregnenolone and progesterone production by the hAmMSCs. **(C)** No difference was observed in the mRNA levels of progesterone-producing factors when SF-1 and WT1−KTS were co-expressed compared with those when SF-1 was expressed alone. Data are presented as the mean ± SD from three independent experiments. *, *p <*0.05; **, *p <*0.01; ***, *p <*0.001. hAmMSCs, human amniotic membrane-derived mesenchymal stem cells; GFP, green fluorescent protein; WT1, Wilms’ tumor 1; SF-1, steroidogenic factor 1; PCR, polymerase chain reaction; SD, standard deviation; KTS, lysine, threonine, and serine.

SF-1 markedly increased the mRNA expression of *StAR*, *CYP11A1*, and *HSD3B2* in hAmMSCs (**Figure 5A**). SF-1 also significantly enhanced pregnenolone and progesterone production by hAmMSCs (**Figure 5B**). In contrast, WT1+KTS and WT1−KTS did not alter the mRNA expression of progesterone-producing factors (**Figure 5A**) nor pregnenolone and progesterone production in hAmMSCs (**Figure 5B**). No difference was observed in the mRNA levels of progesterone-producing factors when SF-1 and WT1−KTS were co-expressed compared with those when SF-1 was expressed alone (**Figure 5C**).

### 3.5 Effects of SF-1 and WT1 on adrenal steroidogenic cell lineage

**Figure 6:**
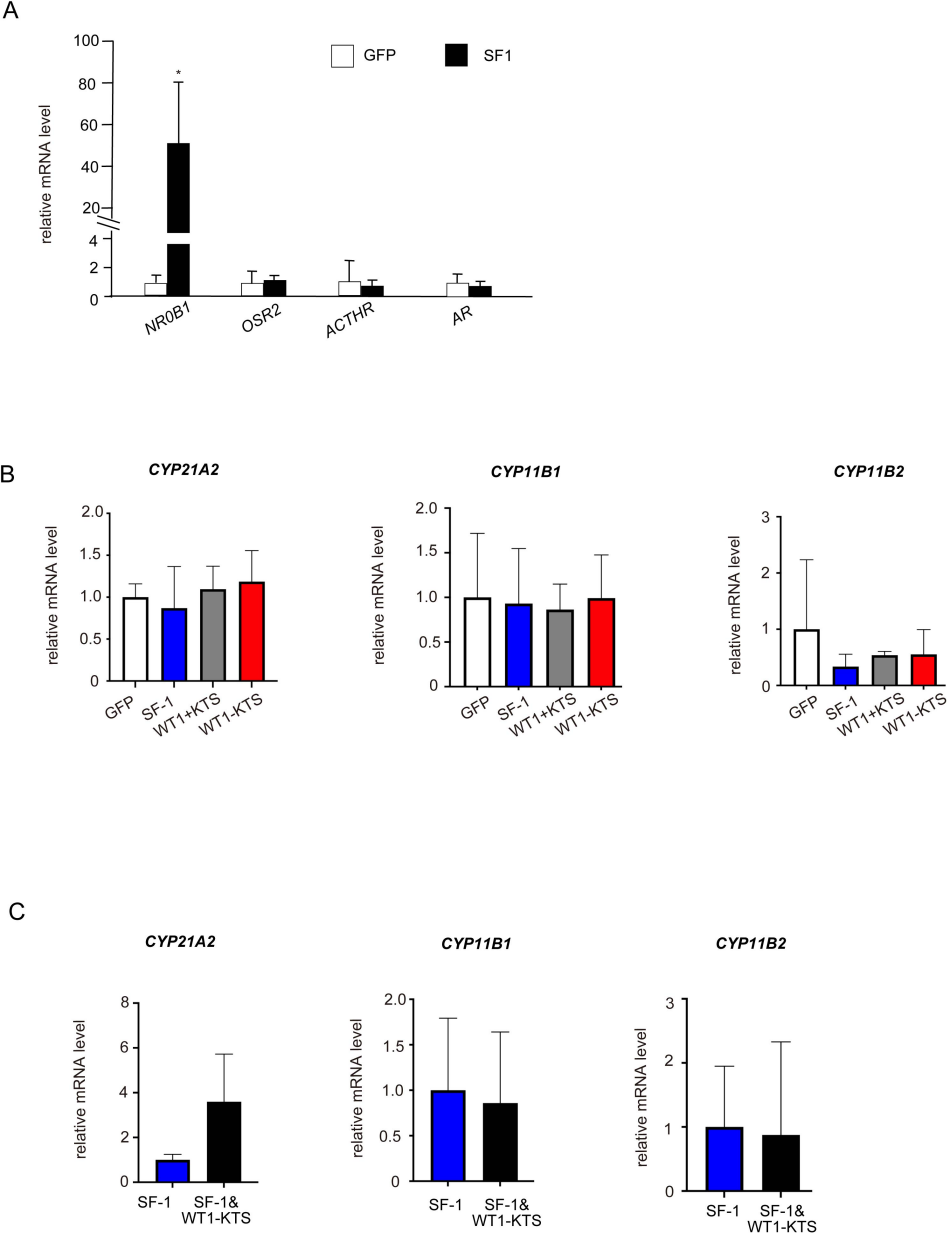
Effects of SF-1 and WT1 on adrenal steroidogenic cell lineage. **(A)** SF-1 significantly increased the mRNA expression of *NRDB1* (*DAX-1*) but not of *OSR2*, *ACTHR*, and *AR*, in hAmMSCs. **(B)** SF-1, WT1+KTS, and WT1−KTS did not change the mRNA levels of *CYP21A2*, *CYP11B1*, and *CYP11B2*. **(C)** Co-expression of SF-1 and WT1−KTS did not alter the mRNA levels of adrenal steroidogenic enzymes in hAmMSCs. Data are presented as the mean ± SD from three independent experiments. *, *p <*0.05. hAmMSCs, human amniotic membrane-derived mesenchymal stem cells; WT1, Wilms’ tumor 1; SF-1, steroidogenic factor 1; SD, standard deviation; KTS, lysine, threonine, and serine.

SF-1 significantly increased the mRNA expression of *NR0B1* but not of *OSR2*, *ACTHR*, or *AR* in hAmMSCs (**Figure 6A**). SF-1, WT1+KTS, and WT1−KTS did not change the mRNA levels of *CYP21A2*, *CYP11B1*, and *CYP11B2* (**Figure 6B**) or cortisol and aldosterone production in hAmMSCs (**Supplementary Figure 1A**). No difference in the mRNA levels of adrenal steroidogenic enzymes was observed when SF-1 and WT1−KTS were co-expressed compared with those when SF-1 was expressed alone (**Figure 6C**).

### 3.6 Effects of SF-1 and WT1 on androgen-producing cell lineage

**Figure 7:**
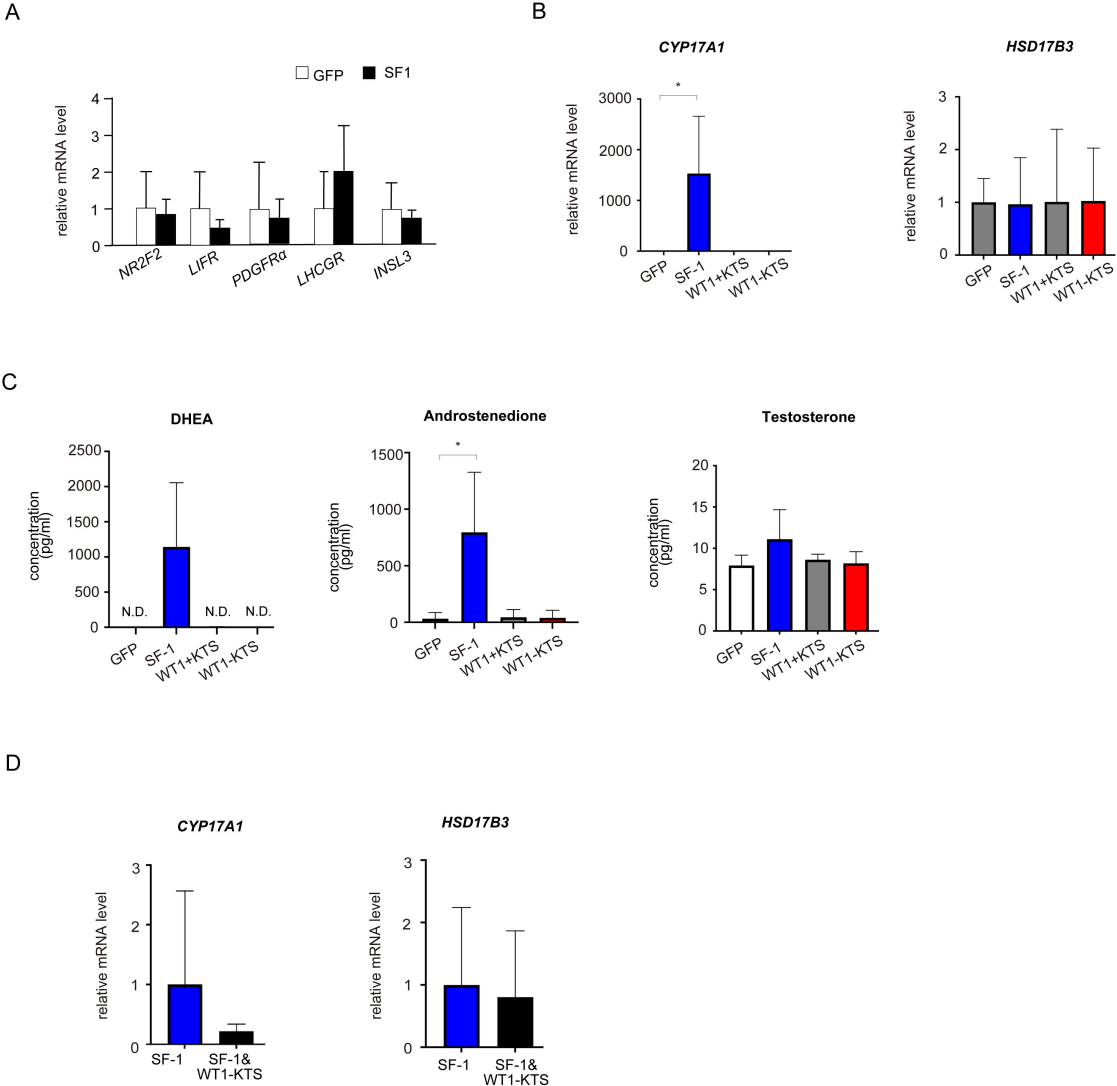
Effects of SF-1 and WT1 on androgen-producing cell lineage. **(A)** SF-1 did not change the mRNA levels of *NR2F2*, *LIFR*, *PDGFRα*, and *LHCGR*. **(B)** SF-1 significantly increased the mRNA expression of *CYP17A1* in hAmMSCs. In contrast, WT1+KTS and WT1−KTS did not alter the mRNA expression of androgen-producing enzymes. **(C)** SF-1 enhanced DHEA and androstenedione production, but not testosterone production, by hAmMSCs. **(D)** No difference was observed in the mRNA levels of androgen-producing factors when SF-1 and WT1−KTS were co-expressed compared with those when SF-1 was expressed alone. Data are presented as the mean ± SD from three independent experiments. *, *p <*0.05; N.D., not detectable. hAmMSCs, human amniotic membrane-derived mesenchymal stem cells; WT1, Wilms’ tumor 1; SF-1, steroidogenic factor 1; DHEA, dehydroepiandrosterone; SD, standard deviation; KTS, lysine, threonine, and serine.

Although SF-1 did not change the mRNA levels of *NR2F2*, *LIFR*, *PDGFRα*, *LHCGR*, and *INSL3* (**Figure 7A**), it markedly increased the mRNA expression of *CYP17A1* in hAmMSCs (**Figure 7B**). SF-1 also enhanced DHEA and androstenedione production, but not testosterone production, by hAmMSCs (**Figure 7C**). In contrast, WT1+KTS and WT1−KTS did not alter the mRNA expression of androgen-producing enzymes (**Figure 7B**) and androgen production in hAmMSCs (**Figure 7C**). No difference was observed in the mRNA levels of androgen-producing enzymes when SF-1 and WT1−KTS were co-expressed compared with those when SF-1 was expressed alone (**Figure 7D**).

### 3.7 Effects of SF-1 and WT1 on estrogen-producing cell lineage

**Figure 8:**
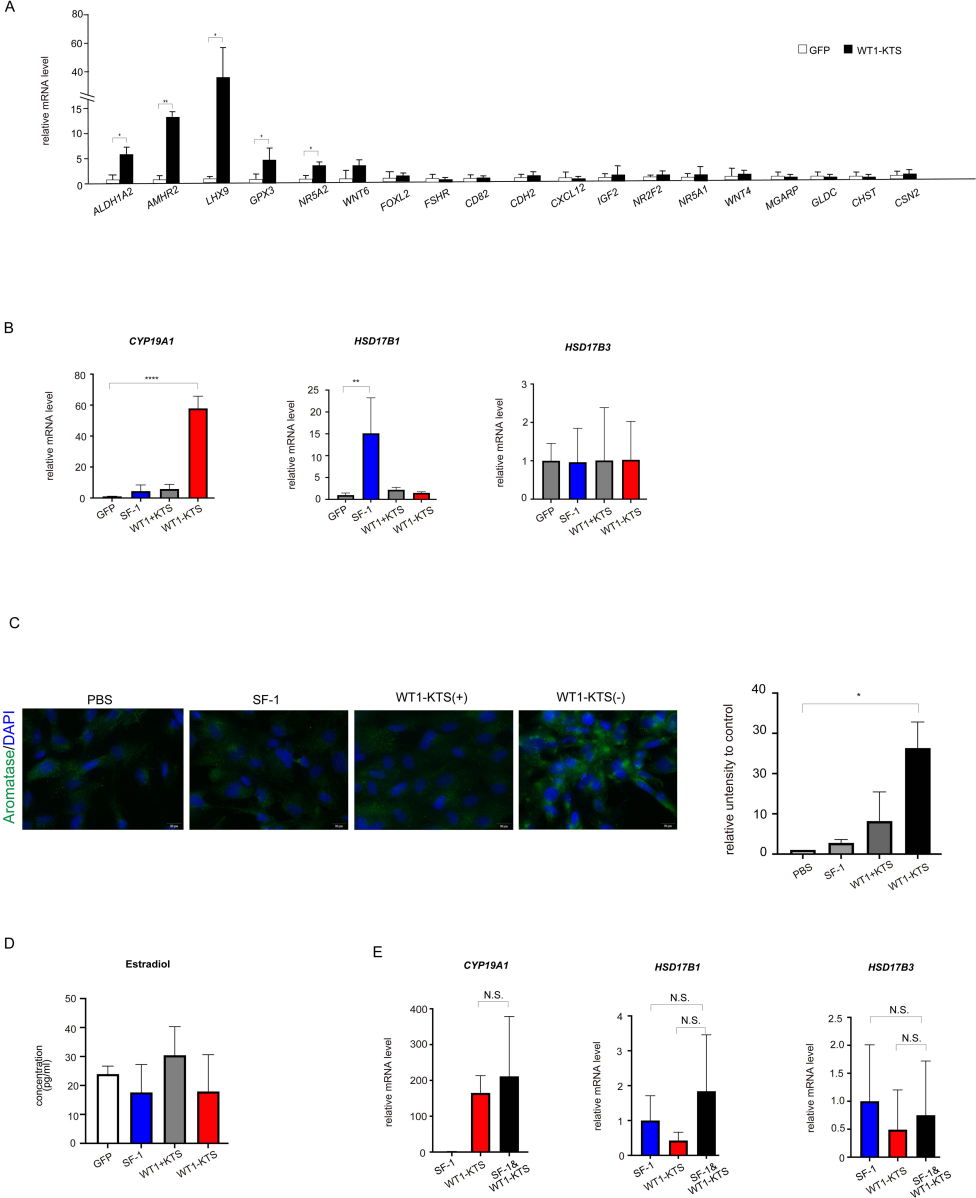
Effects of SF-1 and WT1 on estrogen-producing cell lineage. **(A)** WT1−KTS significantly increased the mRNA expression of *ALDH1A2*, *AMHR2, LHX9, GPX3*, and *NR5A2* in hAmMSCs. **(B)** WT1−KTS, but not WT1+KTS or SF-1, significantly increased the mRNA expression of *CYP19A1* in hAmMSCs. SF-1 increased the mRNA level of *HSD17B1*. **(C)** Immunocytochemical analysis confirmed that WT1−KTS increased aromatase expression in hAmMSCs. **(D)** WT1−KTS and SF-1 did not increase estrogen production by hAmMSCs even in the presence of testosterone. **(E)** Co-expression of SF-1 and WT1−KTS also did not alter the mRNA levels of estrogen-producing enzymes in hAmMSCs. Data are presented as the mean ± SD from three independent experiments. *, *p <*0.05; **, *p <*0.01; ****, *p <*0.0001; N.S., not significant. hAmMSCs, human amniotic membrane-derived mesenchymal stem cells; WT1, Wilms’ tumor 1; SF-1, steroidogenic factor 1; KTS, lysine, threonine, and serine.

WT1−KTS, but not WT1+KTS or SF-1, significantly increased the mRNA expression of several granulosa cell marker genes (*ALDH1A2*, *AMHR2*, *LHX9*, *GPX3*, *NR5A2*) and *CYP19A1* in hAmMSCs (**Figures 8A, B**). Immunocytochemical analysis confirmed that WT1−KTS increased aromatase expression in hAmMSCs (**Figure 8C**). Nevertheless, WT1−KTS did not increase estrogen production by hAmMSCs even in the presence of testosterone (**Figure 8D**). SF-1 increased the mRNA level of *HSD17B1* but did not stimulate estrogen production in hAmMSCs (**Figures 8B, D**). No difference in the mRNA levels of estrogen-producing enzymes was observed when SF-1 and WT1−KTS were co-expressed compared with those when SF-1 was expressed alone (**Figure 8E**).

### 3.8 Activation of CYP19A1 PII promoter by WT1–KTS

**Figure 9:**
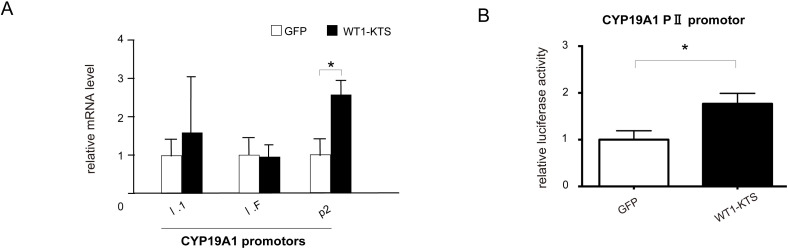
Activation of CYP19A1 PII promoter by WT1–KTS. **(A)** Tissue-specific promoters of human CYP19A1. The mRNA expression of I.1 (placenta), I.f (brain), and PII (ovary) transcripts was observed in hAmMSCs. Transient expression of WT1−KTS significantly increased the mRNA level of the ovary-specific PII promoter in hAmMSCs. **(B)** Ovary-specific PII promoter of human CYP19A1. Luciferase assay revealed that WT1−KTS activated the CYP19A1 PII promoter. Data are presented as the mean ± SD from three independent experiments. *, *p*<0.05. hAmMSCs, human amniotic membrane-derived mesenchymal stem cells; WT1, Wilms’ tumor 1; KTS, lysine, threonine, and serine.

Among the tissue-specific promoters of human CYP19A1, mRNA expression of I.1 (placenta), I.f (brain), and PII (ovary) transcripts was observed in hAmMSCs (**Figure 9A**). Transient expression of WT1−KTS significantly increased the mRNA level of the ovary-specific PII promoter in hAmMSCs (**Figure 9A**). Activation of the CYP19A1 PII promoter by WT1−KTS was also confirmed using luciferase assay (**Figure 9B**).

## 4 Discussion

**Figure 10:**
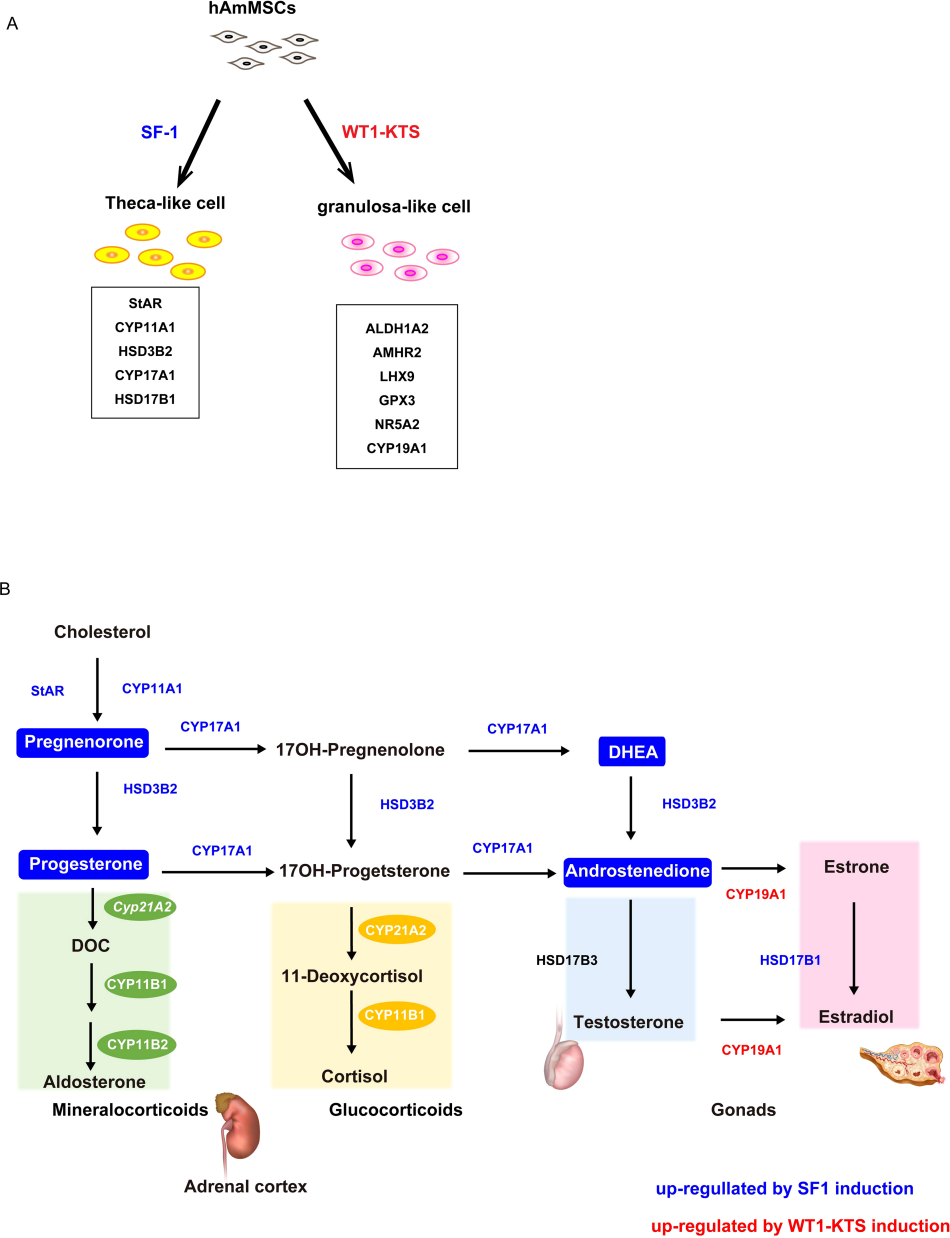
Schema of the hAmMSC differentiation and human steroid hormone biosynthetic pathways. **(A)** Schematic illustration of the differentiation of hAmMSCs into steroidogenic cells. **(B)** Schematic diagram of the human steroid hormone biosynthetic pathway. Steroidogenic enzymes shown in blue and red were upregulated by SF-1 and WT1–KTS induction, respectively. Steroid hormones in the boxes marked in blue were produced by SF-1 induction.

To investigate the role of *SF-1* and *WT1* in the differentiation of human adrenal or gonadal steroidogenic cells, we transiently expressed these genes and investigated their effects on the adrenal and gonadal steroidogenic potential of hAmMSCs. We found that SF-1 and WT1 did not alter the mRNA expression of adrenal steroidogenic enzymes and the production of adrenal steroid hormones in hAmMSCs. In contrast, SF-1 increased the mRNA levels of progesterone-producing factors and progesterone production in hAmMSCs. SF-1 also increased the mRNA levels of androgen-producing enzymes and androgen production in hAmMSCs. The −KTS splice variant of WT1 (WT1−KTS) increased mRNA expression of some granulosa cell markers and ovary-specific aromatase in hAmMSCs; however, it did not induce estrogen production. These results suggest that SF-1 and WT1−KTS play important roles in the differentiation of ovarian steroidogenic cells in humans. In our experimental model, SF-1 directed hAmMSCs to the progesterone- and androgen-producing cell lineage (e.g. ovarian theca cells), whereas WT1−KTS directed hAmMSCs to the estrogen-producing cell lineage (e.g. ovarian granulosa cells) (**Figures 10A, B**).

Several attempts have been made to induce differentiation of ovarian somatic cells using transcription factors (23, 24). Gregoire et al. showed that WT1−KTS is essential for initiating pre-granulosa cell differentiation in mice XX embryos. In addition, WT1−KTS induced male-to-female sex reversal in mice XY embryos. Loss of WT1−KTS blocked gonadal differentiation, whereas its increased expression induced precocious differentiation of the ovaries, independent of the sex of the mice (14). These results suggest that WT1−KTS may be an ovary-determining factor in female mice. It is unknown whether WT1−KTS is also involved in ovarian determination in humans. Nevertheless, it is worth noting that an altered ratio of KTS splice isoforms (i.e. WT1−KTS↑/WT1+KTS↓) causes Frasier syndrome, which is characterized by progressive glomerular nephropathy and male-to-female sex reversal in humans (10, 25, 26). In the present study, WT1−KTS induced the expression of ovary-specific aromatase in hAmMSCs. These findings suggest that WT1−KTS is at least partly involved in the differentiation of ovarian granulosa cells in humans.

Gonadal somatic cell differentiation requires timely and precise coordination among diverse transcription and growth factors (14). Pierson Smela et al. showed that overexpression of SF-1 and Runt-related transcription factor (RUNX) 1/2 differentiated human induced pluripotent stem cells (hiPSCs) into mature granulosa-like cells. The hiPSC-derived granulosa-like cells expressed several granulosa marker genes, formed ovarian follicle structures, and produced progesterone and estradiol (27). In our experimental model, SF-1 induced the differentiation of hAmMSCs to progesterone- and androgen-producing cells but not to estrogen-producing cells. hAmMSCs appear to exhibit lower multipotency compared with that of hiPSCs and demonstrate limited ability to differentiate into mature cells. Even with the combination of SF-1 and RUNX1/2, it would be difficult for hAmMSCs to differentiate into granulosa-like cells similar to hiPSCs.

Chen et al. showed that WT1 directed the lineage specification of Sertoli and granulosa cells by repressing SF-1 expression in mice. Deletion of WT1 upregulated SF-1 expression and induced the differentiation of gonadal somatic cells into Leydig and theca cells, but not into Sertoli and granulosa cells, in mice (7). However, in the present study, WT1−KTS did not suppress SF-1 expression in hAmMSCs. Co-expression of WT1−KTS did not alter SF-1-induced gene expression or steroid production in hAmMSCs. Further investigation is required to determine whether the absence of observable interaction between WT1 and SF-1 is due to species differences between mice and humans or to limitations of our hAmMSC system.

WT1−KTS is expressed not only in XX but also in XY gonads. In mice XY embryos, SRY acts before WT1−KTS, thus promoting testis development and repressing ovary formation in XY gonads (28). If WT1−KTS is upregulated, such as in Frasier syndrome, WT1−KTS antagonizes SRY expression, resulting in pre-granulosa cell differentiation and male-to-female sex reversal in mice (14). In the present study, we were unable to examine the interaction between WT1 and SRY because we primarily used female-derived hAmMSCs without SRY expression. Future studies are required to determine whether it is possible to analyze the regulatory mechanism of SRY by WT1−KTS using male-derived hAmMSCs expressing SRY.

We isolated MSCs from human amniotic membranes for the following two reasons. First, Sasaki et al. showed that the primordial germ cells of cynomolgus monkeys are derived from the amnion (29). Given the similarities in early developmental mechanisms between cynomolgus monkeys and humans, amniotic membranes may also be involved in gonad formation and steroidogenic cell differentiation in humans. The other reason is the high isolation efficiency of hAmMSCs. Generally, the estimated frequencies of MSCs in adult bone marrow and umbilical cord blood are 0.01% and 0.0003%, respectively (30). Pregnancy-associated tissues, such as amniotic membranes and umbilical cords, are rich sources of MSCs and contain higher numbers of MSCs than those in adult tissues such as the bone marrow, peripheral blood, adipose tissue, and muscle (31). hAmMSCs offer several advantages, including noninvasive sample collection, minimal ethical concerns, no age-related heterogeneity, and better proliferative and differentiation potential than that of adult MSCs (31).

The MSC markers CD105, CD90, and CD73 vary widely in terms of expression depending on the organ of origin. For example, approximately 4% of amnion-derived MSCs were CD105-positive, whereas 58% of liver-derived MSCs were CD105-positive (32). Furthermore, CD105 expression decreases with differentiation (33, 34), which may explain the low percentage observed. In this study, only 4% of the cell population was considered positive for CD90, CD105, or CD73, which are MSC markers. The remaining 96% of the cells most likely represent a heterogeneous mix of cells that do not express these MSC markers or may express only one or two of them. Although hAmMSCs may not be as pluripotent as hiPSCs or human embryonic stem cells, they remain useful as low-cost and relatively easily available experimental tools.

## 5 Conclusion

In summary, SF-1 directed hAmMSCs to the progesterone- and androgen-producing cell lineage (e.g. ovarian theca cells), whereas WT1−KTS directed hAmMSCs to the estrogen-producing cell lineage (e.g. ovarian granulosa cells). However, in our experimental model, transient gene transfection with SF-1 and WT1 failed to fully differentiate hAmMSCs into mature steroidogenic cells. In addition to SF-1 and WT1−KTS, a variety of genes and growth factors must be involved in the steroidogenic differentiation of gonadal somatic cells in a complex temporal and spatial manner. This study paves the way for future research on human gonadal development. Further optimization of our hAmMSC system is required to elucidate the mechanism by which human gonads acquire steroidogenic ability.

## Data Availability

The raw data supporting the conclusions of this article will be made available by the authors, without undue reservation.

## Supplementary material

Supplementary Figure 1 Effects of SF-1 and WT1 on adrenal steroidogenic cell lineage. SF-1, WT1 +KTS, and WT1−KTS did not alter cortisol and aldosterone production in hAmMSCs.
