# Supplementary material for: Steroidogenic differentiation of human amniotic membrane-derived mesenchymal stem cells into a progesterone-/androgen-producing cell lineage by SF-1 and an estrogen-producing cell lineage by WT1−KTS
Source: Front Endocrinol (Lausanne). 2024 Sep 18;15:1410433. doi: 10.3389/fendo.2024.1410433 (PMC11445051; doi:10.3389/fendo.2024.1410433)
Supplement: Supplementary file 1 [file Table1.docx]

|  | **Inter assay %CV** | **Intra assay %CV** |
| --- | --- | --- |
| Cholesterol | 17.6 | 18.7 |
| Pregnenolone | 8.6 | 7.0 |
| Progesterone | 20.6 | 7.2 |
| Cortisol | 10.2 | 1.38 |
| Testosterone | 15.2 | 7.6 |
| DHEA | 4.7 | 9.0 |
| androstenedione | 18.3 | 2.4 |
| aldosterone | 18.9 | 11.6 |
| estradiol | 11.4 | 5.3 |

**Supplementary Table 1**

The intra-assay and inter-assay coefficients of variation (CV) of each steroid hormones.
