# Supplementary figures and images for: Steroidogenic differentiation of human amniotic membrane-derived mesenchymal stem cells into a progesterone-/androgen-producing cell lineage by SF-1 and an estrogen-producing cell lineage by WT1−KTS
Source: Front Endocrinol (Lausanne). 2024 Sep 18;15:1410433. doi: 10.3389/fendo.2024.1410433 (PMC11445051; doi:10.3389/fendo.2024.1410433)

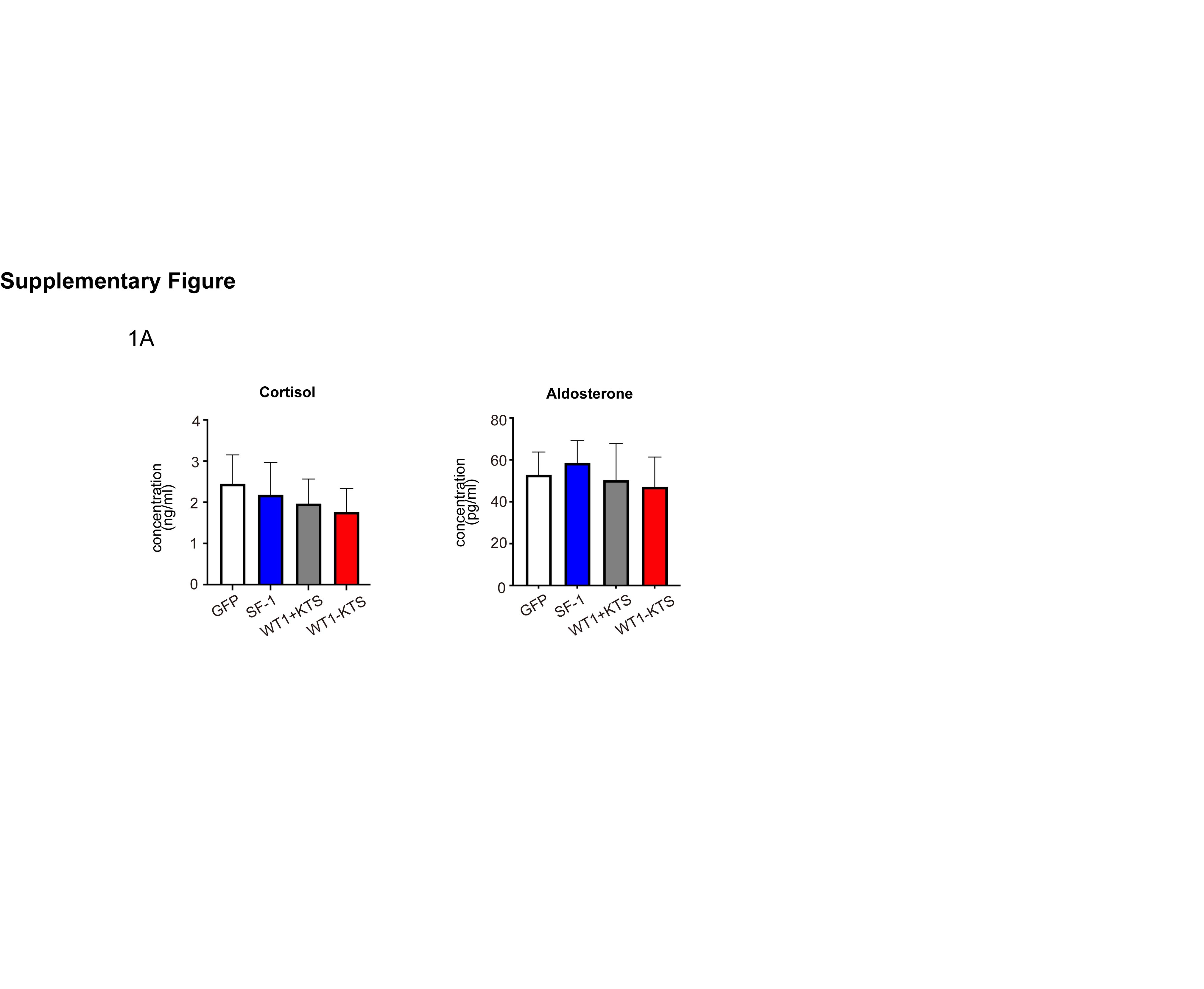

Supplement: Supplementary Figure 1 — Effects of SF-1 and WT1 on adrenal steroidogenic cell lineage. SF-1, WT1 +KTS, and WT1−KTS did not alter cortisol and aldosterone production in hAmMSCs. [file Image1.jpg]
